# Neurological disorders in Pregnancy: Radiological insights and Obstetric implications

**DOI:** 10.12669/pjms.41.1.9680

**Published:** 2025-01

**Authors:** Rukhsana Aziz, Sumaira Yasmin, Nasreen Aman, Huma Shams

**Affiliations:** 1 Rukhsana Aziz, FCPS Department of Radiology, Medical Teaching Institute, Lady Reading Hospital, Peshawar, Pakistan; 2 Sumaira Yasmin, FCPS Department of Obstetrics and Gynaecology, Medical Teaching Institute, Lady Reading Hospital, Peshawar, Pakistan; 3 Nasreen Aman, FCPS Department of Radiology, Medical Teaching Institute, Lady Reading Hospital, Peshawar, Pakistan; 4 Huma Shams, MBB Department of Obstetrics and Gynaecology, Medical Teaching Institute, Lady Reading Hospital, Peshawar, Pakistan

**Keywords:** MRI, Fetal outcome, Neurological disorders, Posterior reversible encephalopathy syndrome (PRES), Dural sinus thrombosis (DST)

## Abstract

**Objective:**

To explore the radiological findings of neurological disorders in obstetrics patients, their obstetric and fetal outcome.

**Method:**

The cross-sectional study was conducted at Lady Ready Hospital (LRH), Peshawar from June 2022 till March, 2023. Sixty two obstetric patients with neurological symptoms were included. Their clinical records were explored. MRI/MRV brain were performed in patients requiring brain imaging and followed till the end of pregnancy.

**Results:**

Seizures were the commonest symptom found in 74.2% patients followed by hemiparesis; 8.1%. About 92% had never experienced hypertension and 62.3% had pregnancy-induced hypertension in current pregnancy. Twenty eight patients underwent MRI, revealing Dural sinus thrombosis (DST) in 42.9%, posterior reversible encephalopathy syndrome (PRES) in 17.9%, infarcts in 14.3% and hemorrhage in 7.1%. Normal baby was born in 69% patients; stillbirth, preterm birth and miscarriage were found in 25.9%, 3.2% and 1.6% respectively. Apgar scores of 7–10, 4-6 and 0-3 were found in 67.2%, 6.9% and 25.9% respectively. Fits were observed in 10.3% babies and 32.8% required neonatal intensive care unit (NICU) care. Fits and ischemic infarct were commoner in prenatal. DST and PRES were commoner in postnatal patients. All PRES patients presented with fits. DST patients presented with fits, headache, hemiparesis and altered consciousness. All PRES patients delivered normal alive babies. DST mothers delivered alive babies at term in 81.8%, dead pre-term baby and miscarriage in 9.1% each.

**Conclusion:**

Pregnancy-related neurological problems should be taken carefully and evaluated, since even seemingly insignificant symptoms like headaches may be signs of serious illnesses like PRES or DST.

## INTRODUCTION

Neurological disorders (NDs) in pregnancy have a diverse presentation like migraine, epilepsy, hypertension-related complications including eclamptic fits, drug induced peripheral neuropathies, tumors and many others. The diagnosis and management of the NDs in pregnancy are always a challenging task due to varied symptomatology and risks to the fetus.^1^ Minor NDs are often overlooked in pregnancy. Some mononeuropathies like carpel tunnel syndrome and facial palsy are much prevalent in pregnancy but the severity of symptoms and functional impairment are mild.^2^,^3^

The mortality rate and burden of disability caused by NDs are higher than any other major disease worldwide.^4^ A few NDs have serious complications for mother with impact on fetus and neonate. Most of these conditions have similar clinical presentations. The cause may be diagnosed in some cases with imaging techniques especially using MRI, while others may have no radiological finding. Thus, we tried to find out the spectrum of these varied neurological manifestations in pregnancy, correlated clinical manifestations with radiological findings and evaluated their obstetric outcome. NDs are at large, preventable, treatable and curable, if properly diagnosed and treated. Effective preventive and therapeutic strategy could not only decrease the disease burden in society but also reduce the disability.^5^

## METHODS

The cross-sectional study was carried out from June 2022 to March 2023 at the Lady Reading Hospital (LRH), Peshawar in Radiology and Obstetrics and Gynecology departments. Sixty two obstetric patients who presented to Gynecology Department were enrolled in the study. Simple convenient sampling technique was used. Non pregnant Patients with neurological disorder or lost to follow up before the end of pregnancy were excluded from study. Using the OpenEpi version 3.01 algorithm, the sample size was determined by setting the incidence of pregnancy-related stroke at 30 per 100,000, as reported in a study.^6^

### Ethical Approval:

The study was approved by the institutional ethics review committee’s with Ref. (407/LRH/MTI) Dated June 14, 2022.

Patients clinical records were explored for risk factors, neurological symptomatology, gestational age and any associated comorbidities (hypertension. diabetes, cardiac and any other) were entered on proforma. Informed consent was taken from patients/attendants and they were followed in Gynecology Department till the end of pregnancy. patients in all trimesters of pregnancy were included in the study and also followed postnatally for one week. Those who required patients brain imaging were referred to radiology department for an MRI brain. Those neurological disorders which did not require brain imaging were not subjected to it. Patients requiring MRI were referred to radiology department and imaged using MRI Machine (1.5 Tesla, Toshiba). DWI was employed along with standard MRI brain and MRV (Magnetic Resonance Venography) imaging techniques. Two consultant radiologists evaluated every MRI scan. Data was evaluated using SPSS V26. Continuous those variables were reported as means ± standard deviation and categorical variables as frequencies/percentages.

## RESULTS

**Fig.1:**
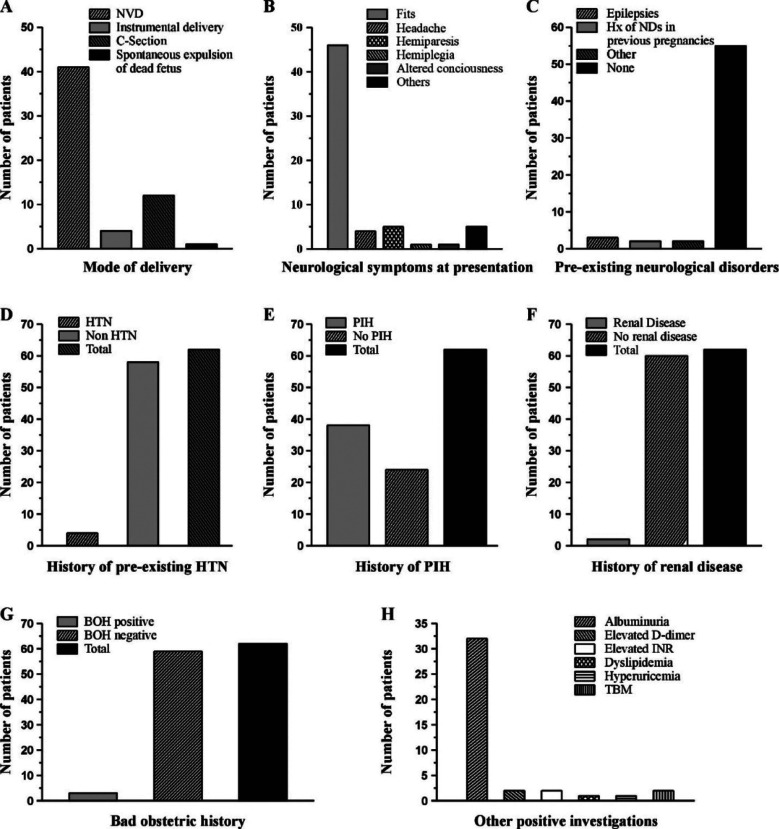
Clinical and laboratory data of patients. A; Mode of delivery, B; Neurological symptoms of presentation, C; Pre-existing neurological disorders, D; History of preexisting hypertension, E; History of PIH, F; History of renal disease, G; Bad obstetric history, H; Other positive investigations.

There were 62 patients having a mean age of 25.66 ± 5.92 years (range: 15-45 years). 24 patients (38.7%) were postnatal, and 38 patients (61.3%) were antenatal. Among antenatal patients, most were primigravidae (n=20; 52.63%) followed by multigravidas i.e. G2-G4 (n=13; 34.21%). Furthermore, neurological symptoms were found even in grand gravidas i.e. G5 and above (n=5; 13.16%). Majority of postnatal patients were primiparas (13 out of 24; 54.17%) followed by multiparous patients i.e., 11; 45.83%. Thirty-four (89.5%) and four (10.5%) antenatal patients presented in third and second trimester of pregnancy respectively while none of them presented in first trimester. One-third of postnatal patients presented during 1st week of delivery (8; 33.3%), although patients presented even up to 45th postnatal day (7^th^ week). Forty-one patients (70.7% of the total) delivered via normal vaginal delivery while 12 (20.7%) patients had undergone cesarean section (Fig.1A). Seizures were the commonest presentation found in 46 (74.2%) patients followed by hemiparesis in 5 (8.1%) patients.

**Fig.2:**
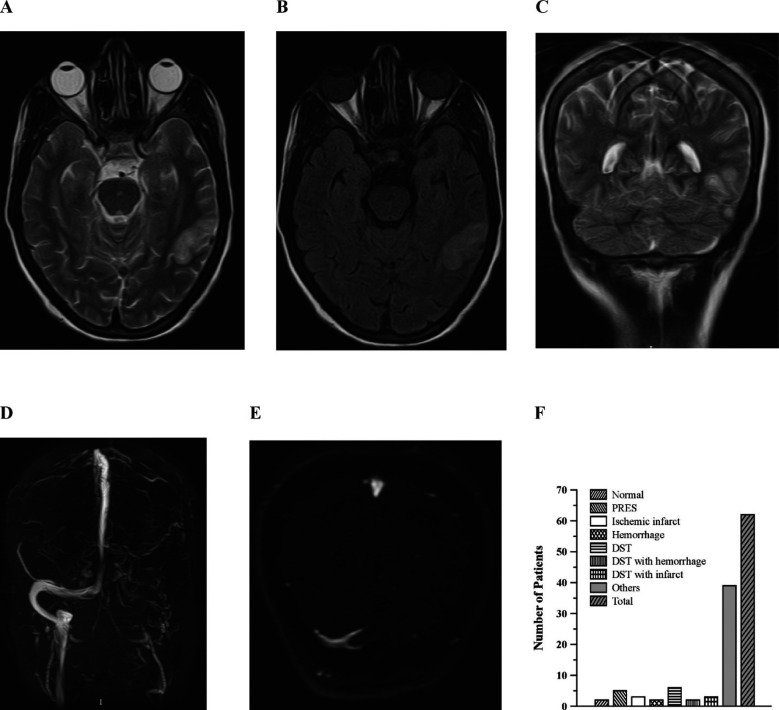
Findings of MRI Brain. A; T2W axial image, B; FLAIR axial image both showing hyper intense area in left temporal lobe, C; T2W coronal image showing left temporal lobe hyper intensity and loss of signal void in left transverse sinus, D; 3D VR MRV and E; Axial MRV reveals non visualization of left transverse & sigmoid sinus as well as internal jugular vein with evidence of multiple collateral vessels confirming left transverse and sigmoid sinus thrombosis with temporal lobe infarct, F; graphical presentation of various MRI findings.

Five patients (8.1%) had additional neurological symptoms, such as sore hands, tremors, numbness and tingling in the hands and feet, irregular gait, and visual impairment. In four cases (6.5%), the presenting complaint was headache. One patient (1.6%) each had altered consciousness and hemiplegia (Fig.1B). Fifty five (88%) patients had no history of prior neurological illnesses. Two patients (3.2%) had a history of neurological issues in previous pregnancy, and three patients (4.8%) had a history of epilepsy (Fig.1C). Fifty seven patients (91.9%; Fig.1D) had no history of pre-existing hypertension. Pregnancy-induced hypertension (PIH) was present in 38 (62.3%) patients during their current pregnancy (Fig.1E). One patient (1.6%) had a history of diabetes mellitus. Two patients (3.2%) each had renal disease and bad obstetric history (Fig.1F&G). A disrupted lipid profile, increased uric acid in one patient (1.6%) each, high D-dimers and INR in two patients (3.2%) each, and albuminuria in 50% of patients were among the additional positive tests. Abnormal nerve conduction tests were found in two (3.2%) patients (Fig.1H). Among 28 patients, who underwent MRI brain, 12 (42.9%) had dural sinus thrombosis (DST), followed by posterior reversible encephalopathy syndrome (PRES) in 5 (17.9%), ischemic infarcts in 4 (14.3%) and hemorrhage in 2 (7.1%). Meningoencephalitis (n=2) and AV malformation (n=1) were among the others. Two (7.1%) had normal MRI brain scans (Fig.2). All patients were managed conservatively.

**Fig.3:**
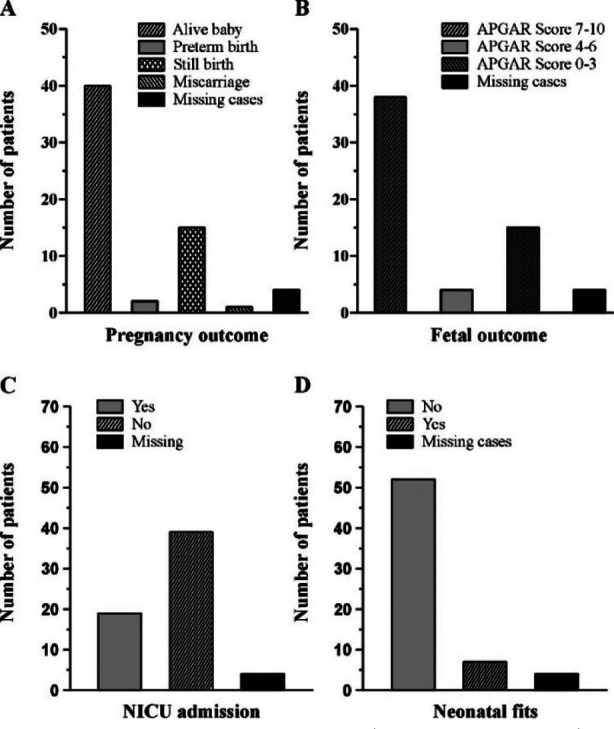
Pregnancy & fetal outcome. Bar graphs represent pregnancy outcome (A), fetal outcome (B), NICU admission of newborns (C), and neonatal fits (D).

Four patients were lost of follow up. So, pregnancy outcome could be found in 58 patients that revealed delivery of normal alive baby in 40 (69%) followed by still birth in 15 (25.8%), preterm birth in 2 (3.4%) and miscarriage in 1 (1.7%) patient (Fig.3A). Fetal outcome was evaluated in terms of Apgar score; 7-10 score was found in 38 (66.7%), 4-6 in 4 (7%) and 0-3 in 15 (26.3%) babies (Fig.3B). No congenital anomaly was found in any fetus. Nineteen (32.8%) babies needed neonatal intensive care unit (NICU) care while six had fits in neonatal period (10.3%; Fig.3C&D).

**Fig.4:**
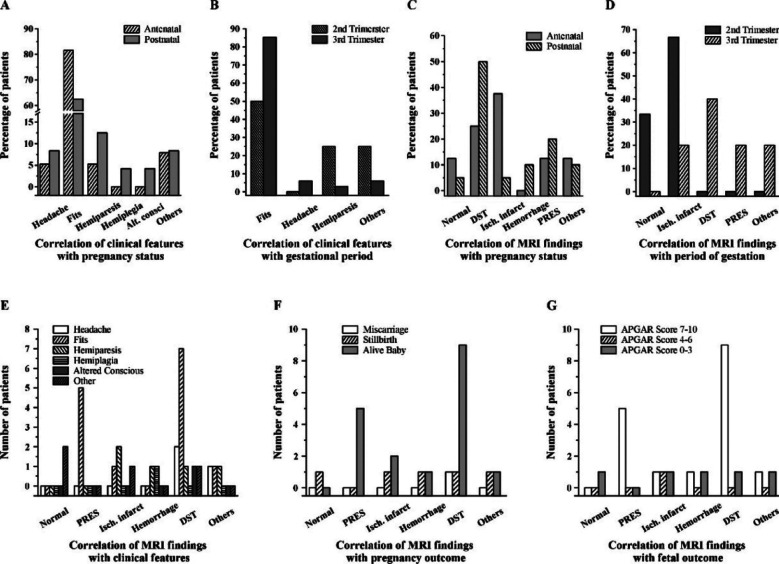
Correlation of clinical features of patients with pregnancy status (A), with trimester-wise period of gestation (B), correlation of MRI finding with pregnancy status (C), with period of gestation (D), with clinical features (E), with pregnancy outcome (F) and with fetal outcome of patients (G).

Fits were more common in antenatal than postnatal patients, with 81.5% vs. 62% (Fig.4A) and 85% vs. 50% in the third trimester compared to the second (Fig.4B). Hemiparesis was more common in second trimester—25% vs. 2.9%—than in the third (Fig.4B). While ischemic infarct was more common in antenatal than postnatal patients—37.5% vs. 5%— Fig.4C shows that DST and PRES were more common in postnatal —50% and 20%, respectively—than in antenatal patients—25% and 12.5%, respectively. While 66.6% of patients with ischemic infarct were discovered in their second trimester, DST and PRES were more common in the third trimester; none of the patients in the second trimester had these symptoms (Fig.4D). All of the PRES patients had fits, whereas 58.3% of the DST patients had fits, 16.6% had headaches, 8.3% had hemiparesis, altered consciousness, and other symptoms each. Half of the participants with an ischemic infarct had hemiparesis, 25% had fits and 25% had other symptoms; on the other hand, 50% of the subjects with a hemorrhage had hemiparesis and hemiplegia (Fig.4E).

While 81.8% of patients with DST delivered healthy babies at term, 9.09% delivered dead preterm babies, and 9.09% experienced miscarriages, all patients with PRES delivered alive babies with an Apgar score of 7–10 (Fig.4F). Hence, 90% of infants born to DST women had an Apgar score of 7 -10 (Fig.4G).

## DISCUSSION

Gravidity and neurological state are correlated bilaterally because physiological changes that occur during pregnancy might alter the course of some disorders by making them less severe or more severe.^7^ In this study, a significant proportion of antenatal patients (90.6%) showed symptoms in the third trimester, while only 9.4% presented in second trimester. This data raises the possibility that neurological problems during pregnancy are more frequently linked to later gestational stages or become more noticeable during those stages. The commonest neurological symptom, affecting 74.2% of patients, was fits. These results comply with study in which epilepsy was the commonest ND with incidence 207/100000 pregnancies.^8^ Women with epilepsy had an increased risk of complications related to pregnancy and delivery like preterm delivery, cesarean delivery, and low birth weight.^9^,^10^

Hemiparesis was another neurological illness manifestation in 8.1% of patients. Moreover, 8.1% of individuals experienced symptoms like tingling, numbness, tremors, and vision impairment. The primary complaint in 6.5% of instances was headache which is comparable to our previous study.^11^ However, a few studies documented higher frequency of headache.^12^,^13^ This difference may have arisen because of the low socioeconomic status of presented population, or a lack of awareness about the importance of headaches, which prevents individuals from obtaining radiological testing until there is a severe neurological issue. Notably, among individuals with NDs, the prevalence of PIH in the current pregnancy is considerable (62.3%). This study emphasizes the possible link between PIH and neurological abnormalities, and it is imperative to manage hypertension during pregnancy to lower the risk of unfavorable outcomes. This is in line with published data.^11^,^14^,^15^

Hyperintensity on FLAIR images in the parietooccipital and posterior frontal cortical and subcortical white matter is the most prevalent imaging result associated with PRES; the brainstem, basal ganglia, and cerebellum are less frequently affected.^16^ Altered sensorium, visual disturbances, status epilepticus and elevated serum uric acid, lactate dehydrogenase, and creatinine are associated with abnormal neuroimaging findings.^13^,^16^ MRI brain scans mainly showed DST and PRES, hemorrhage and infarcts. Meningoencephalitis and AV malformation were also identified in a single case each. The range of radiological results points to the possibility that several etiological variables could be involved in the development of neurological symptoms during pregnancy. These findings provide valuable insight into the pathophysiology of neurological disorders during pregnancy and can guide healthcare providers in selecting appropriate diagnostic imaging modalities and treatment strategies. The highest percentage of DST and PRES is similar to studies conducted in Asian populations.^11^,^17^,^18^ DVST is believed to be more common in poor countries than in high income countries because of the higher frequency of poor nutrition, infections and dehydration.^18^ DST is linked to potentially deadly illnesses like hemorrhagic stroke and is the cause of 6% of maternal deaths.^19^

Pregnancy-related DST is most common in the third trimester and puerperium. Since early ischemic alterations in individuals with acute NDs cannot be found on CT scans, MRI is considerably more effective in diagnosing these changes than CT scanning. Furthermore, on MRI DWI images, PRES can be distinguished from well-developed infarcts. The infarct exhibits diffusion restriction and looks bright on DWI images and dark on ADC Map, however both states appear hypodense on CT scans and hyper intense on T2W/FLAIR MRI images. On DWI in PRES, no brightness is observed. On MRV, dural sinuses can be easily assessed without the need for contrast, and a thrombus can be seen as a filling defect inside the typically bright-looking sinuses. Consequently, MRI alone or with MRV will be the preferred radiological investigation. Patients with early signs of neurological problems should not be disregarded, and an appropriate assessment should be conducted.

The results of pregnancy outcome highlight the possible gravity of neurological disease during pregnancy and its potential harm to the developing fetus. Regarding fetal outcome in patients with neurological disorders, adverse outcome was common in these patients. It might not be solely due to the neurological disorder in itself, but it might be due to associated complications. While a significant proportion of patients delivered normal, healthy babies (69%), a substantial number experienced intrauterine fetal death or adverse neonatal outcomes. Stillbirths including intrauterine fetal deaths (IUFDs) and intrapartum deaths were 29.8%. In evaluating fetal well-being, APGAR scores revealed that the majority of babies (67.2%) had scores between 7-10, indicating good overall health at birth. However, a significant proportion (25.9%) had lower APGAR scores (below 3), warranting closer attention to neonatal care in these cases.

These findings emphasize the need for close monitoring of both maternal and fetal well-being throughout the pregnancy. The variations in APGAR scores highlight the challenges in managing neonates born to mothers with NDs, indicating the need for immediate medical attention and neonatal intensive care. This underscores the importance of neonatal resuscitation and specialized care for infants born to high-risk mothers. A poor fetal outcome could be due to inductions or termination of pregnancy in patients whose condition was deteriorating due to eclampsia. However, further research is needed to establish specific cause-effect relationships between different neurological disorders and adverse fetal outcomes. In addition, this study does not include data on the frequency of neurological disorders in the general population so that we can find any increase frequency of certain neurological disorders in our obstetric population as compared with general population.

## CONCLUSION

It is concluded and recommended that careful antenatal monitoring of blood pressure is needed, especially in third trimester. More vigilance should be observed in addressing neurological symptoms in pregnancy and postpartum period to prevent adverse maternal and fetal outcomes. A holistic approach involving collaboration between obstetrician, radiologist and neurologist is essential to better manage these conditions.
